# Supplementary material for: Poly(2-Hydroxyethyl methacrylate-co-N,N-dimethylacrylamide)-Coated Quartz Crystal Microbalance Sensor: Membrane Characterization and Proof of Concept
Source: Gels. 2021 Sep 24;7(4):151. doi: 10.3390/gels7040151 (PMC8544454; doi:10.3390/gels7040151)
Supplement: Supplementary file 1 [file gels-07-00151-s001.zip › gels-1352579-supplementary.pdf]

gels

MDPI

Article

Poly(2-Hydroxyethyl methacrylate-co-N,N -dimethylacrylamide)-Coated Quartz Crystal Microbalance Sensor: Membrane Characterization and Proof of Concept

Angel Ramon Hernandez-Martinez

Centro de Física Aplicada y Tecnología Avanzada, Universidad Nacional Autónoma de México (UNAM), Boulevard Juriquilla 3001, Queretaro 76230, Queretaro, Mexico; angel.ramon.hernadez@gmail.com or arhm@fata.unam.mx

Supplementary information

Swelling Measurements

The swelling ratio (Sr) is a relative measure that quantifies the mass or volume gain of hydrogel membranes (HMs) due to water adsorption onto the polymeric network. The gavimetric method was the procedure followed to evaluate %Sr, for which cylindrical samples of approximately 100 mg of dry hydrogels were used. They were then immersed in glass vials containing distilled water, the vials were previously placed at a controlled temperature of 298.15 K using a temperature-controlled water bath. Afterwards, at regular time intervals, the HMs were removed from the vials to record their weight and immediately returned to their respective vials. This was repeated n times until at least 5 identical weights were recorded. In this way, a series of data on weight variation with respect to time of immersion in distilled water was obtained for each HMs and from this data %Sr was calculated according to the following equation:

$$Sr = \frac{Ms - Md}{Md} \tag{1}$$

Ms and Md are the mass of swollen and dry hydrogel respectively; and theThe water adsorption profiles (swelling ratio, Sr) for each copolymer at different time intervals are shown in Fig. S1; in each profile the equilibrium point is indicated.

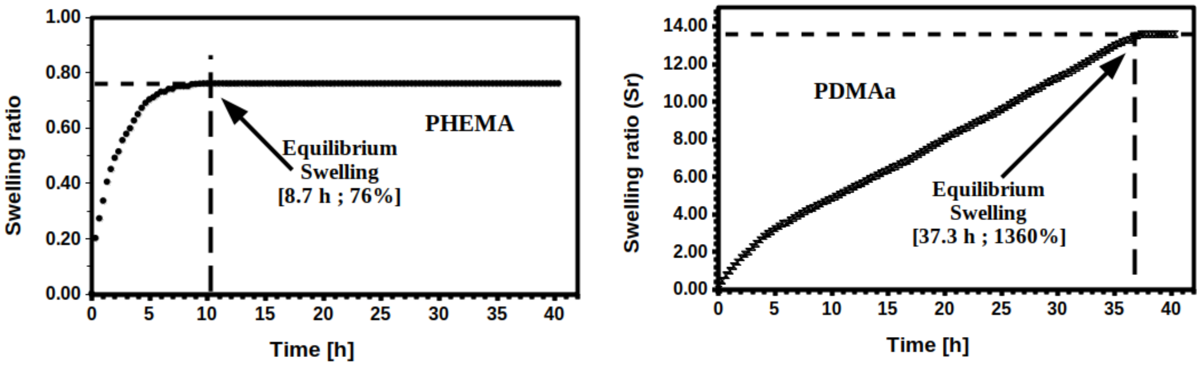

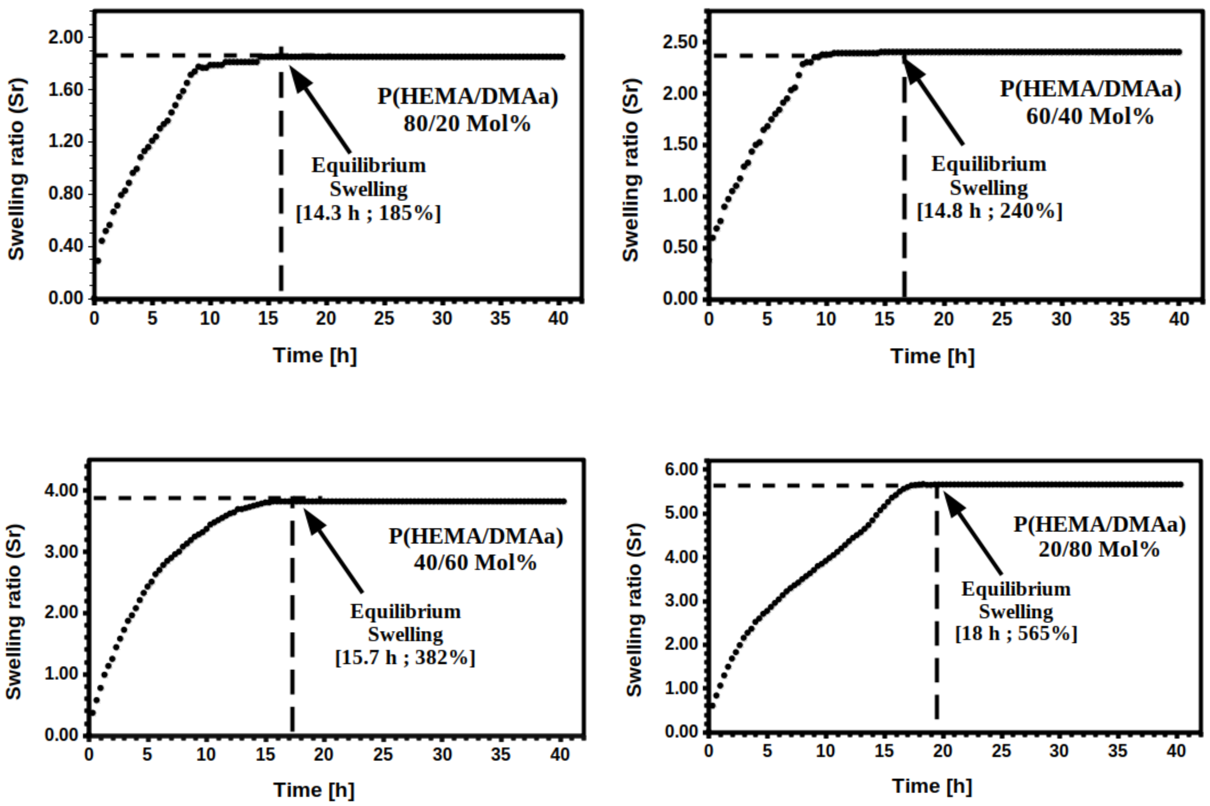

Figure S1. Swelling profiles at 298.15 K and pH 7 of the p(HEMA-co-DMAa) copolymers.

Sorption Studies

Ion adsorption was investigated from aqueous solutions in batch experiments. pH (pH = 7.5; adjusted by dropwise addition of NaOH conc) and temperature (298.15 K) of the medium were fixed variables to study the effects of initial concentration on adsorption rate and adsorption capacity as a function of varying mol% content of DMAa. The lowest initial concentration was 10 ppm, while the highest was 300 ppm in all cases. The amounts of adsorbed ions per unit mass of adsorbent at any time t (Qt, mg × g^{-1}) and at equilibrium (Qe, mg × g^{-1}) were calculated by using the following equations:

$$Q_{t[mg*g^{-1}]} = \frac{(C_i - C_t)}{m_{(g)}} \quad (2)$$

$$Q_{e[mg*g^{-1}]} = \frac{(C_i - C_e) * V_{(L)}}{m_{(g)}} \quad (3)$$

where Ci and Ce are initial and equilibrium ions concentrations (mg × L^{-1}; or ppm) respectively; Ct is ions concentration at any time t; V is the volume of the solution added (L) and m is the amount used of hydrogel (g). Ct was determined by Uv-vis spectroscopy at different time intervals until Ce was reached, i.e. a constant final concentration.

The variation of the amounts of adsorbed ions per unit mass of adsorbent (Qt) as a function of time showed a behaviour as shown in figures S2a and S2b. Figure S2a shows Qt versus time for the initial concentration (Ci) of 40 ppm lead ions, while Fig S2b shows the case for a Ci of 80 ppm. In both cases a maximum Qt or plateau is reached; this point represents the physicochemical equilibrium, where the adsorption and desorption rate is the same, so that maximum Qt remains constant over time. Equilibrium concentration (Ce), mounts of adsorbed ions per unit mass of adsorbent at equilibrium (Qe), and the time required to reach equilibrium (Te) are obtained at this physicochemical equilibrium;

these are parameters of the equilibrium reached for a series of system conditions such as temperature (298.15 K), pH (7.5), and initial concentration, which was variable. According to figures S2 a and b, Ci is a variable that influences the physicochemical equilibrium of the adsorbent/adsorbate system; while for an initial concentration of 40 ppm a Qe of 31 mg Pb(II) per gram of adsorbent was achieved; for a Ci of 80 ppm a Qe of 39 mg Pb(II) per gram of adsorbent was achieved; in both cases the copolymer with 50 mol% DMAa was used.

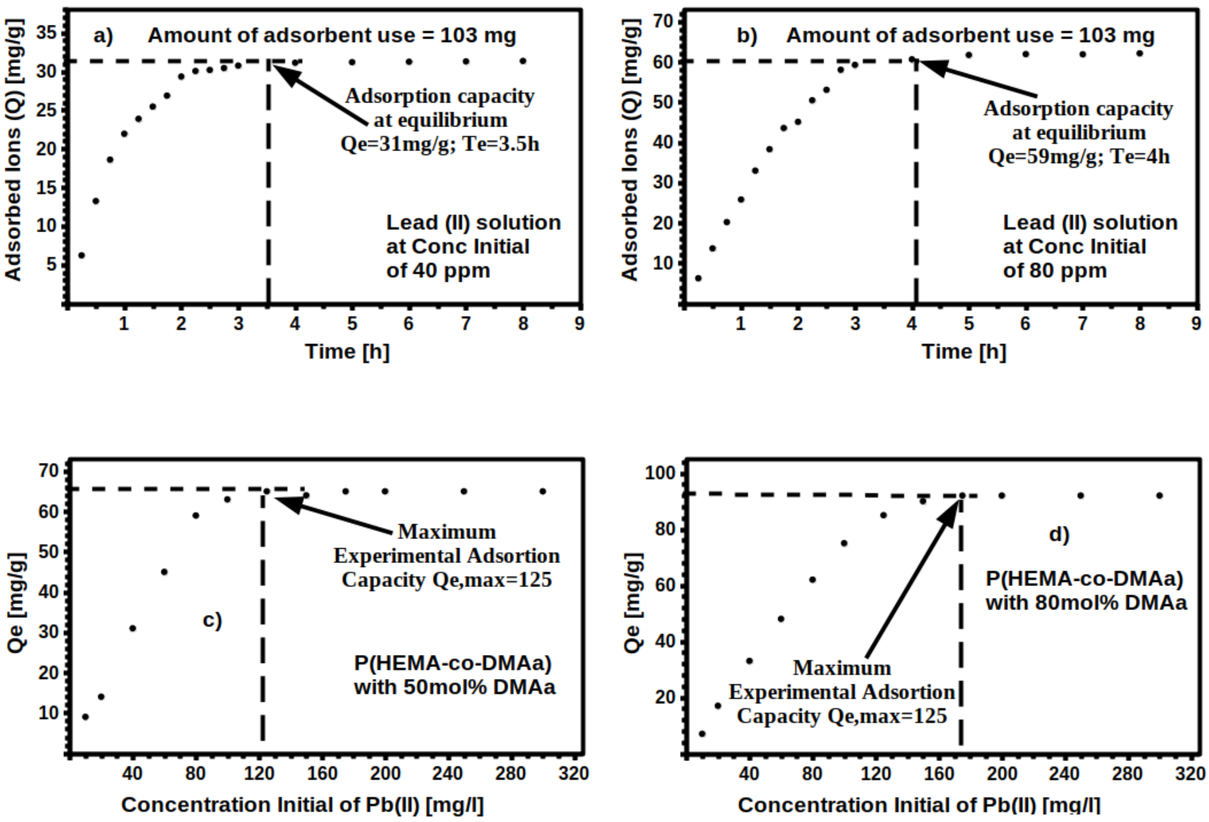

Figure S2. Amounts of adsorbed lead ions per unit mass of adsorbent (Qt) as function of time at 298.15 K and pH 7.5 to **a)** concentration initial of 40 ppm and **b)**80 ppm, using p(HEMA-co-DMAa) copolymer whit 50 mol% content of DMAa.Qe versus Ci for **c)** p(HEMA-co-DMAa) copolymer whit 50 mol% content of DMAa and **d)** p(HEMA-co-DMAa) copolymer whit 80 mol% content of DMAa.

For this same copolymer, figure S2c shows the complete picture, Qe as a function of initial concentration (Ci) shows that more Pb(II) is adsorbed at higher initial concentrations; up to a limiting value, where Qe remains constant regardless of an increase in initial concentration. For the case of the copolymer with 50 mol% DMAa, the maximum Qe is achieved at a Ci of 125 ppm. Figure S2d shows the case of the copolymer with 80 mol% DMAa and in this case the maximum Qe is achieved with a Ci of 175 ppm.

Therefore, the parameters Qe, Ce and Te were similarly determined at different Ci, for each copolymer with different DMAa content. Thus, Figure S3 shows the Qe profile as a function of Ci for all copolymers in solutions of lead(II); copper(II); cobalt(II); cadmium(II); chromium(III); and iron(III).

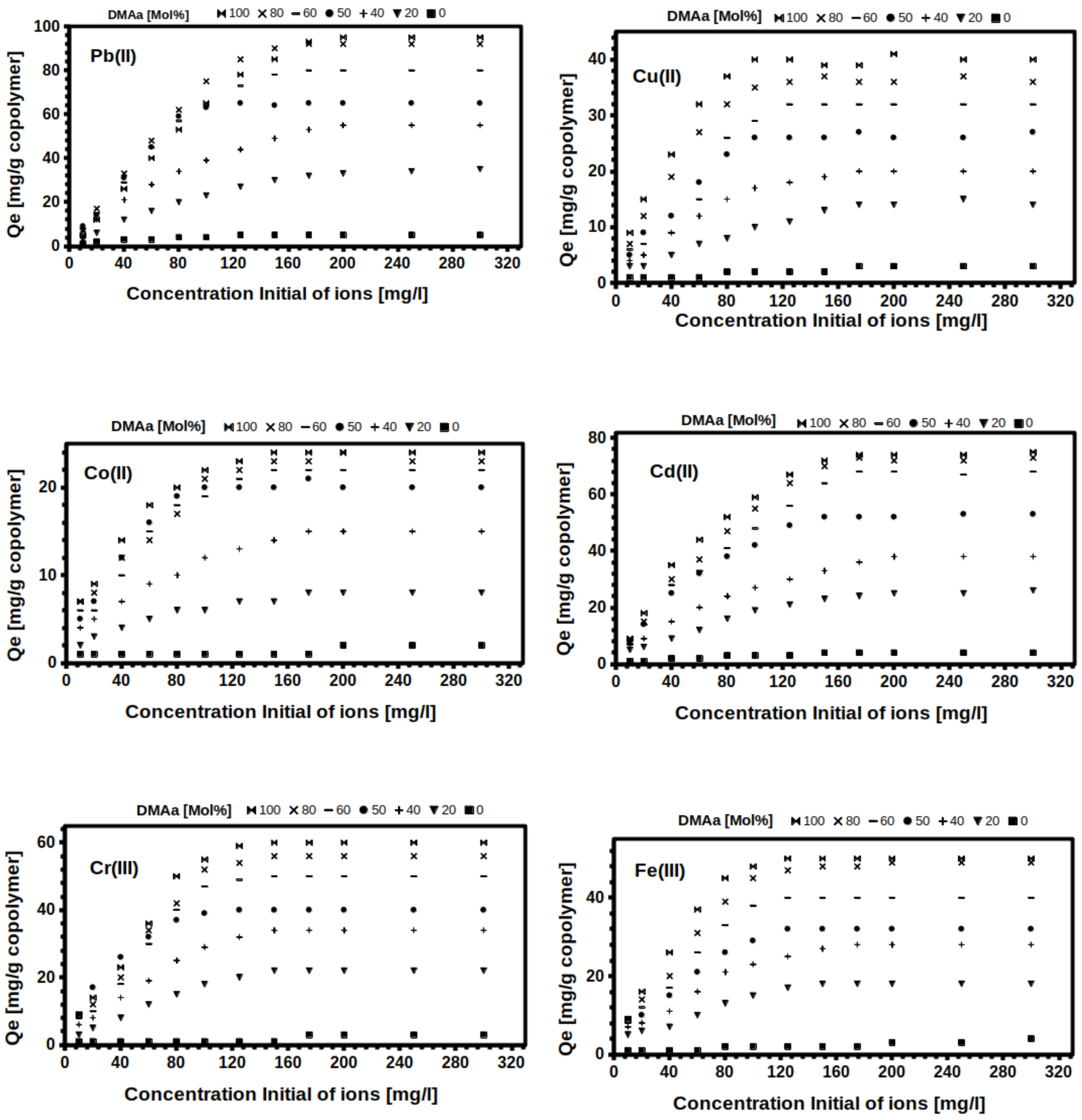

Figure S3. Adsorption isotherm; Qe versus Ci profiles, at 298.15 K and pH 7.5 of all p(HEMA-co-DMAa) membranes for each of the ions; Pb(II), Cu(II), Co(II), Cd(II), Cr(III), and Fe(III).
